# Xylem Parenchyma Anatomy and Gene Expression Patterns Indicate Mechanisms of Cavitation Resistance in *Eucalyptus grandis* During Drought

**DOI:** 10.1002/pei3.70068

**Published:** 2025-06-21

**Authors:** Rafael Keret, Paul N. Hills, David M. Drew

**Affiliations:** ^1^ Department of Genetics, Institute for Plant Biotechnology Stellenbosch University Stellenbosch South Africa; ^2^ Department of Forestry and Wood Sciences Stellenbosch University Stellenbosch central South Africa

**Keywords:** embolism, hydraulic safety, transcriptome, vessel associated cells, wood anatomy

## Abstract

Tree survival under drought conditions depends on the ability to maintain functional xylem and avoid hydraulic failure due to cavitation. Although xylem parenchyma are key sites of metabolic activity in angiosperm wood, the anatomical and gene expression responses of this cell type to drought stress remain poorly characterized. This study investigated how *Eucalyptus grandis* W. Hill ex Maiden modifies parenchyma anatomy and gene expression under water deficit to enhance cavitation resistance. Under controlled drought conditions, plants produced significantly smaller ray parenchyma cells with increased frequency. This arrangement reduced the proportion of isolated vessels through elevated ray‐vessel contacts, likely enhancing solute delivery to stressed conduits. Transcriptomic analysis revealed upregulation of aquaporins, lipid transfer proteins, and enzymes involved in triacylglycerol biosynthesis, supporting roles in water transport and nanobubble stabilization under negative pressure. In parallel, genes associated with osmotic regulation, including various sugars, myo‐inositol, and metal ion transporters, were also induced, indicating putative solute‐mediated mechanisms for refilling embolized vessels. This transcriptomic response appears to be primarily triggered by oxidative and hypoxic stress signals. Collectively, these results indicate that xylem parenchyma contribute to embolism resistance by actively redistributing water and supporting hydraulic stability during drought. This work provides mechanistic insights into tree drought adaptation, with implications for forest management and climate resilience strategies.

## 1 Introduction

Given the uncertainties with future climate and the effects this may have on trees planted in diverse environments, understanding drought responses and adaptation is incredibly valuable to meet the world's insatiable hunger for timber products (Rudel et al. 2020; Tabari 2020). Several tree species have been adopted for commercial forestry, but among them, *Eucalyptus* has emerged as a global success, owing to excellent wood properties and adaptability to strenuous conditions (Mostert‐O'Neill et al. 2022; Seng Hua et al. 2022; Myburg et al. 2014). Although these adaptations have enabled eucalypts to survive extreme climates, this is also a caveat for timber industries, since abiotic stressors, especially water availability, drastically affect the commercially valuable wood properties (Pfautsch et al. 2016; Câmara et al. 2020; Lourenço et al. 2022). In particular, xylem parenchyma displays a remarkable variability within the angiosperm lineage and typically comprises a considerable portion of the wood, ranging from 7% to 64%, which is largely dependent on the tree provenance and species (Morris et al. 2018). However, the degree of plasticity of these cell populations within the xylem of *Eucalyptus* is understudied and may serve as a crucial mechanism for tree survival.

The mature secondary xylem of plant stems comprises mainly tracheary elements, fibers, and parenchyma (Eckert et al. 2019). Tracheids occurring in gymnosperms and vessels in angiosperms serve as the main water conductive elements in the wood, with fibers further providing mechanical support (Rodriguez‐Zaccaro and Groover 2019). Considering the essential roles of tracheary elements and fibers, the function of xylem parenchyma in wood is an often overlooked aspect of tree biology but is gaining research interest (Słupianek et al. 2021). The functional life cycle of parenchyma does not involve programmed cell death in the same way as those destined to be tracheary elements. Hence, parenchyma is a living cell fraction of the functional xylem (Kotowska et al. 2020). In angiosperms, ray and axial parenchyma form major interconnected networks that bridge the phloem and xylem, thus enabling the exchange of water and solutes throughout the stem (Tomasella et al. 2020). Specialized parenchyma, termed vessel‐associated cells, occur at the interface of these networks to form direct linkages to vessels through pit membranes or pores (Secchi et al. 2017). Overall, these living cells are responsible for the storage or transport of water, carbohydrates, lipids, and ions that are critical for tree survival during periods of abiotic stress (Słupianek et al. 2021). Although still a topic of ongoing debate, parenchyma is emerging as having an important role in hydraulic safety and recovery via several proposed embolism prevention and repair mechanisms (Secchi et al. 2017; Saunders and Drew 2022).

Vessels transport water under negative pressures and are consequently susceptible to cavitation that causes air bubbles to enter the metastable water column (Stroock et al. 2014). Once the tension in the sap reaches a critical threshold, these gas bubbles can rapidly expand to form embolisms and spread through the vascular system, leading to non‐conductive elements (Trifilò et al. 2019). To prevent complete hydraulic failure of the vasculature, trees can respond via stomatal closure to reduce water tension as a short‐term safety mechanism (Martin‐StPaul et al. 2017). However, the associated reduction in CO_2_ uptake will have negative implications for photosynthesis and ultimately result in tree mortality via carbon starvation over a prolonged period (Qaderi et al. 2019). Thus, tree survival requires an intricate balance between the prevention of hydraulic failure and carbon starvation, which operate as non‐mutually exclusive mechanisms (Hammond et al. 2019).

Given the negative implications associated with embolism, trees have devised mitigation strategies that are, in part, facilitated by the living parenchyma cells (Słupianek et al. 2021). One such mechanism involves redirection of the stored water reserves through the ray parenchyma to the conduits, thus effectively reducing the water tension and the probability of air seeding (Pfautsch et al. 2015). This process is partly facilitated by the action of aquaporins that regulate radial water movement across the cellular membrane (Schenk, Jansen, and Hölttä 2021). In *Eucalyptus globulus* , for example, plasma membrane intrinsic protein (*PIP*) *1*;*2/2*;*7* and tonoplast intrinsic protein (*TIP*) *1*;*3* were identified as candidates for embolism avoidance in response to high vapor pressure deficit (Feltrim et al. 2021). Another putative mechanism by which trees maintain hydraulic safety is via the action of surfactants, mainly in the form of phospholipids, galactolipids, and triacylglycerol in the xylem sap (Huang et al. 2023). Although still an emerging topic in the field of tree hydraulics, the delivery of lipids into vessels is supposedly conducted via the vessel‐associated cells, involving the action of non‐specific lipid transfer proteins (LTPs) for transport (Schenk, Michaud, et al. 2021). The lipids in the xylem sap are thought to stabilize nanobubbles, thereby preventing their expansion beyond a critical size to form embolisms (Schenk et al. 2017). This process has been recently coined the “lipid‐nanobubble hypothesis” and is complimentary to the cohesion tension theory by providing clues as to how the coating of nanobubbles in the xylem sap enables conduits to stably transport water under negative pressures (Schenk, Michaud, et al. 2021). Evidently, these embolism‐preventative mechanisms enable trees to function in water‐limited environments, via the reduction of xylem tension, and prevention of embolism expansion in the functional conduits.

Despite these safety nets, water‐limiting conditions can lead to the formation of embolisms in the conduits and will thus require hydraulic recovery mechanisms (Saunders and Drew 2022). Vessel‐associated cells are at the forefront of embolism repair in trees by initially sensing the embolized vessels and subsequently creating an osmotic gradient that favors water movement into the conduits, thereby initiating refilling (Słupianek et al. 2021). Sucrose, derived from localized starch degradation or phloem export, is proposed to accumulate in the apoplast of embolized vessels that can no longer carry these away in the sap stream, thus priming the repair response (Secchi and Zwieniecki 2011). On a molecular level, in *Populus trichocarpa* , these mechanisms have been shown to correspond to the expression of genes associated with the metabolism or transport of carbohydrates, ions, and water (aquaporins), with hypoxic stress and responses to reactive oxygen species (ROS) likely serving to trigger these (Secchi et al. 2011). Similar genetic responses to embolism were observed in grapevine leaf petioles (Perrone et al. 2012). Finally, the accumulation of sugar compounds and ions is proposed to serve as osmotica to drive water flow into embolized vessels, thus facilitating refilling (Tomasella et al. 2020). Principally, transcriptomic studies have proposed the expression of sugar and metal ion transporters in xylem parenchyma as the primary mechanisms for generating osmotic gradients (Secchi et al. 2017).

Although the majority of embolism prevention and repair mechanisms rely on the metabolically active xylem parenchyma, our understanding of the anatomical and metabolic adaptations that occur in these cells during drought is lacking (Słupianek et al. 2021; Secchi et al. 2017), especially in the context of the globally important species, *Eucalyptus grandis* . We hypothesize that drought stress triggers anatomical changes in the xylem parenchyma that increase ray‐vessel connectivity, thereby facilitating water redistribution and enhancing cavitation resistance. Complementary to this, the expression of genes associated with hydraulic safety, osmotic regulation, and water transport may offer insights as to how the sap flow is maintained under negative pressures in drought‐exposed plants. To test these hypotheses, we analyzed the xylem anatomy of droughted *E. grandis* and conducted a differential expression analysis of the stem transcriptome to identify which physiological processes are triggered during drought. Further elucidating the role of xylem parenchyma, the metabolically active fraction of the xylem, is essential for understanding how *E. grandis* manages hydraulic safety and recovery under drought. This knowledge will improve our ability to anticipate tree responses to climate change and support strategies for drought‐resilient forestry. (Schumann et al. 2019).

## 2 Materials and Methods

### 2.1 Study Context

Data reported in this study were collected concurrently during a previous experiment in which a water deficit was imposed on *E. grandis* (Keret et al. 2024), allowing for the extraction of novel insights. The microsection images (Zenodo: https://doi.org/10.5281/zenodo.8246235, https://doi.org/10.5281/zenodo.8245566), transcriptomic pipeline (GitHub: https://github.com/Rafael‐Keret/Eucalyptus_transcriptomic_analysis), and sequence data (NCBI: PRJNA1012834) are freely accessible online.

### 2.2 Experimental System

Rooted cuttings of *E. grandis* were grown in a shade netted area for 6–8 months (−33.926557027282996, 18.867576102025062), in pots containing 1.5 L of palm peat and filter sand (1:1; V/V) with 2 g of Osmocote Pro slow‐release fertilizer (Osmocote, Gauteng, South Africa). Once a height of 45–50 cm was reached, 24 plants were randomly divided into control and drought treatment groups (*n* = 12). These plants were transferred into a growth chamber in a randomized Latin square design and acclimated for 7 days, receiving 180 mL of water daily. On day 8, differential watering was imposed, with droughted plants receiving 60 mL while controls remained at 180 mL. This 24 h transition period preceded an experimental phase of 30 days, which was repeated three times independently (*n* = 36). Soil water content reflectometers (CS655‐L, Campbell Scientific) revealed an average soil moisture of 0.065 m^3^ m^−3^ in droughted pots compared to 0.283 m^3^ m^−3^ in controls, representing an approximate 77% reduction in water availability. The selected duration and intensity of drought were informed by preliminary trials, which demonstrated that physiological drought stress could be imposed in *E. grandis* without halting secondary growth (Keret et al. 2024). This approach ensured measurable anatomical and molecular responses.

Conditions were maintained at an ambient temperature of 25°C ± 1, relative humidity of 75% ± 5.66, with 16 h light and 8 h dark cycles. Photosynthetically active radiation (PAR) was logged (CR1000x, Campbell Scientific, Cape Town, South Africa) at 111 ± 0.8 μmols photons m^−2^ s^−1^ at three quarters canopy height and 50 ± 1 μmols photons m^−2^ s^−1^ in the middle of the canopy, with pyranometers (CS300L, Campbell Scientific). Lighting was provided with Nano LED grow tubes (T8‐900mm, The Lamphouse, Johannesburg, South Africa).

The transpiration rate was measured every second day with a LiCor porometer/fluorometer (LI‐600, Campbell Scientific; Table S2), 1 h into the photoperiod, on the first three fully expanded leaves. Pre‐dawn leaf water potential (Ψpd; Table S3) was measured every fifth day by initially bagging the leaves in Ziploc bags during the dark period for 1 h, and subsequently harvesting these for Ψpd measurement (MPa) using a Skye plant moisture system (Skye instruments Ltd., Llandrindod Wells, United Kingdom).

### 2.3 Histological Processing and Image Analysis

The histological and microscopy practices follow those of Keret et al. (2024). In short, stem samples were cut 30–35 cm from the apical bud of each plant (*n* = 36) and embedded within paraffin wax blocks (411663, Merck, Darmstadt, Germany). The paraffin blocks were transversely sectioned at 6 μm thickness and placed onto microscope slides for staining with Safranin‐Alcian blue (84120 & A5268; Merck). A Nikon eclipse Ni‐E upright motorized microscope (Nikon Corporation, Düsseldorf, Germany) with a Nikon DS‐Fi2 camera, was used to scan the permanent slides at 20× magnification. Once scanned, the bioimage analysis software, QuPath v0.4.4 (Bankhead et al. 2017), was used to directly measure the cell area for the ray and axial parenchyma in both watering regimes (Tables S4 and S5). The proportional cell area (%) of the parenchyma was derived from the QuPath‐generated variables. Finally, the total number of rays, ray‐vessel contacts, and the proportion of isolated vessels (i.e., isolated versus total vessels) within the defined region of interest were also quantified using the same software (Table S6). Isolated vessels are those that are not directly or indirectly in contact with ray parenchyma.

### 2.4 RNA Isolation and Sequencing

RNA extraction, transcriptomic processing, and analysis are detailed in Keret et al. (2024). In summary, 24 plant stems were debarked 25–30 cm below the apical bud to remove the phloem. Thereafter, a sharp scalpel was used to harvest mainly developing xylem tissue and traces of cambium, 2 h before the photoperiod. Each tissue sample was immediately flash‐frozen in liquid nitrogen. The stem material from three plants were pooled to produce four samples per watering regime (*n* = 4). Subsequently, total RNA was isolated using a CTAB‐based protocol (White et al. 2008). The RNA was assessed for integrity using the RNA ScreenTape system (CAF, Stellenbosch, South Africa) and for purity by Qubit assay (CAF).

Eight cDNA libraries were generated at Macrogen (Macrogen Europe, Amsterdam, Netherlands) using the TruSeq Stranded mRNA LT Sample Prep Kit (20020595, Illumina, Europe) and sequenced to an average depth of 58.7 M paired‐end reads (Illumina Novaseq 6000; Macrogen). The quality of the raw sequences was assessed in FastQC (Andrews 2010) and subsequently processed using Trimmomatic v0.32 (Bolger et al. 2014) to remove low‐quality reads and adapters. Hisat2 v2.2.1 (Kim et al. 2019) was implemented to map the high‐quality reads to the *E. grandis* reference genome (https://www.ncbi.nlm.nih.gov/datasets/genome/GCF_000612305.1/) before assembly into a counts table using the package FeatureCounts v2.0.5 (Liao et al. 2014).

### 2.5 Differential Expression Analysis and Gene Functional Annotation

As in Keret et al. (2024), the read counts were subject to normalization and significance testing in the R System for Statistical Computing v4.3.1 (R Core Team 2022) using the DESeq2 package with default parameters (GitHub: https://github.com/Rafael‐Keret/Eucalyptus_transcriptomic_analysis/tree/main/1.DESeq2) (Love et al. 2014). Genes displaying fewer than 5 counts across all samples were removed, yielding a total of 31,415 genes. The ENTREZ IDs were submitted to the NCBI datasets online portal, via the command line tools option, to extract the corresponding coding sequences of the genes. The closest *Arabidopsis thaliana* ortholog was identified by translated nucleotide BLAST (blastx, *e*‐value ≤ 0.001) of the extracted gene models against a locally built ARAPORT11 (https://www.arabidopsis.org/download_files/Proteins/Araport11_protein_lists/Araport11_pep_20220914.gz) protein database (Zhu et al. 2023). These orthologs were used to assign symbols, functional descriptions, and ontology to the *E. grandis* gene IDs in R, by applying the packages biomaRt (Smedley et al. 2009) and clusterProfiler (Wu et al. 2021), respectively (GitHub: https://github.com/Rafael‐Keret/Eucalyptus_transcriptomic_analysis/tree/main/2.Gene_anotation). The non‐model organism feature of clusterProfiler was implemented to perform gene set enrichment analysis (Wu et al. 2021). Lastly, differentially expressed genes were filtered from the dataset on the basis of an absolute log_2_ fold change > 1 and a *p*_adj_ < 0.05. BiomaRt and custom MapMan functional annotation tables were created for the identification of genes potentially involved in hydraulic safety and osmotic regulation.

The literature was explored to identify biological processes and gene expression candidates that are predominantly associated with the metabolically active parenchyma (Słupianek et al. 2021; Tomasella et al. 2020; Secchi et al. 2017; Schenk et al. 2017). This theoretical knowledge was applied to mine the transcriptomic dataset using R search queries (GitHub: https://github.com/Rafael‐Keret/Eucalyptus_transcriptomic_analysis/tree/main/4.Functional_characterization_physiological). Genes involved in the stress response as well as the metabolism and transport of non‐structural carbohydrates, water, lipid surfactants, and ions were targeted from the transcriptome and compared for differential expression.

### 2.6 Statistical Analysis

Statistical analysis, visualizations, and interpretation of the physiological, anatomical, and transcriptomic data were conducted in R v4.3.1 software (R Core Team 2022). Normality was assessed via quantile‐quantile plots and Shapiro–Wilks tests, while the homogeneity of the variance assumption for models was determined using residual versus fit plots (Kozak and Piepho 2018). Datasets that failed to display a Gaussian distribution or homoscedasticity were appropriately log or square root transformed such that a student's t‐test or repeated measures ANOVA could be performed. Tukey's honestly significant difference was selected for *post hoc* analysis. If the transformations failed to generate normal data, a Wilcoxon signed‐rank test was used for significance testing.

## 3 Results

### 3.1 Impact of Drought on Transpiration, Ψpd and Parenchyma Architecture

**FIGURE 1:**
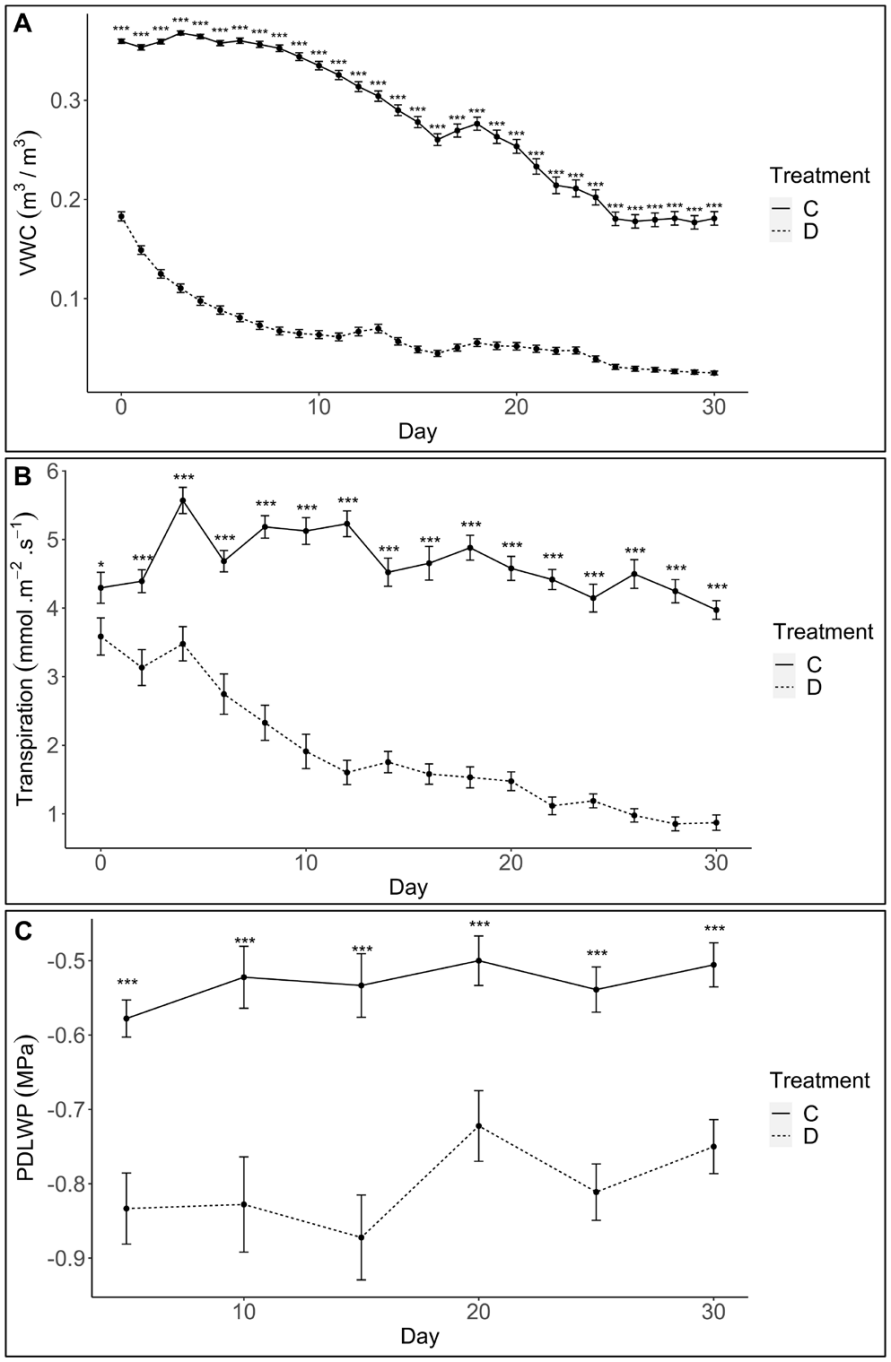
*Eucalyptus grandis* physiological responses to water deficit conditions. (A) The volumetric water content, (B) transpiration and (C) pre‐dawn leaf water potential (Ψpd), measured throughout the 30‐day growth experiment. The treatment key represents the control (C) and droughted (D) conditions tested. Significant differences are depicted as asterisks as follows: *p ≤ 0.01 and ***p ≤ 0.0001.

To investigate the adaptive response in *E. grandis* , water deficit conditions were imposed that appear to have significantly (*p* < 0.01) reduced the soil volumetric water content throughout the growth experiment. Following an initial drastic decline in soil moisture over the first 10 days, a more gradual decline was observed for the remainder of the experiment (Figure 1A). In accordance with the drop in soil water content, the droughted plants strongly regulated their transpiration rate, leading to significantly reduced levels (Figure 1B). This response was not mirrored in the control plants, suggesting that the water content was adequate to maintain high transpiration rates over the experiment. Moreover, the droughted plants displayed significantly (*p* < 0.01) lower water potentials compared to the adequately hydrated controls (Figure 1C). On day 20, a spontaneous upward trend in the Ψpd occurred in the water stressed plants (Figure 1C).

**FIGURE 2:**
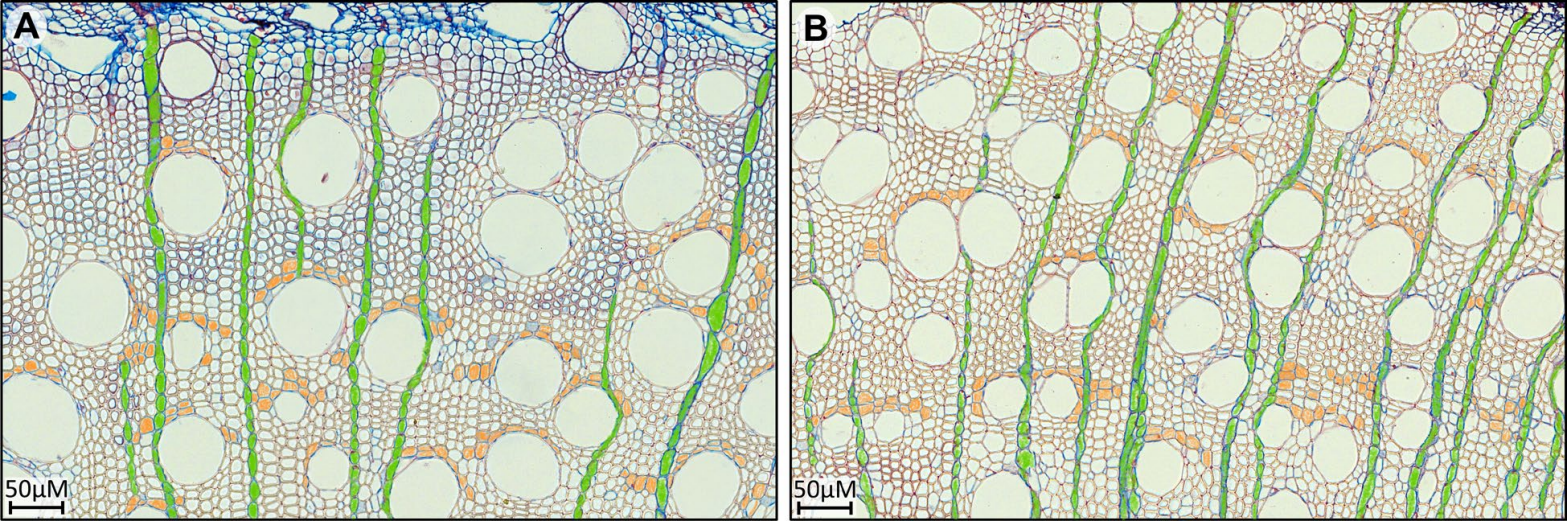
*Eucalyptus grandis* wood anatomical adaptations to (A) control and (B) droughted conditions. The green shaded area indicates instances of ray parenchyma in the wood, whereas the orange shading represents axial parenchyma. The figures clearly display the higher frequency of ray parenchyma in (B) drought‐adapted xylem in *Eucalyptus grandis* . Microsections were stained with Safranin‐Alcian blue and scanned under 20× objective lens.

The drought treatment invoked differences in the parenchyma prevalence and their association with vessels in the xylem (Figure 2A,B). Although the cell area of the axial parenchyma (*p* = 0.17) was unaffected, significantly smaller (*p* = 0.02) ray parenchyma were produced during drought (Table 1). It appears that drought did not influence the overall proportional cell area of the parenchyma in the xylem (*p* = 0.77). Rather, a significant increase (*p* < 0.01) in the number of rays and ray‐vessel contacts was observed (Table 1), suggesting an adaptive response to cavitation risk. These features significantly reduced (*p* = 0.04) the number of isolated vessels from 19.08% in controls to 12.08% during drought.

**TABLE 1:** Wood anatomical adaptations of xylem parenchyma in the stem of *Eucalyptus grandis* subject to control and droughted conditions.

Cell/tissue	Property	Control	Drought	*p*‐value
Axial parenchyma	Cell area (μm^2^)	104.7 (2.42)	102.4 (3.91)	0.17
Ray parenchyma	Cell area (μm^2^)	152.3 (6.09)	134.1 (5.79)	0.02
Stem	Proportional cell area (%)	14.23 (0.83)	14.54 (0.86)	0.77
	Ray number	6.00 (0.27)	7.31 (0.33)	< 0.01
	Ray‐vessel contacts	7.55 (0.43)	11.31 (0.74)	< 0.01
	Isolated vessels (%)	19.08 (2.96)	12.08 (2.43)	0.04

*Note:* The mean measurements of 36 samples per treatment (*n* = 36) are displayed, with the standard error depicted in parenthesis. Data displaying normal distributions were analyzed with a *t*‐test, whereas abnormally distributed data were subject to the Wilcoxon‐sign ranked test.

### 3.2 Transcriptomic Themes Associated With Embolism Response

**FIGURE 3:**
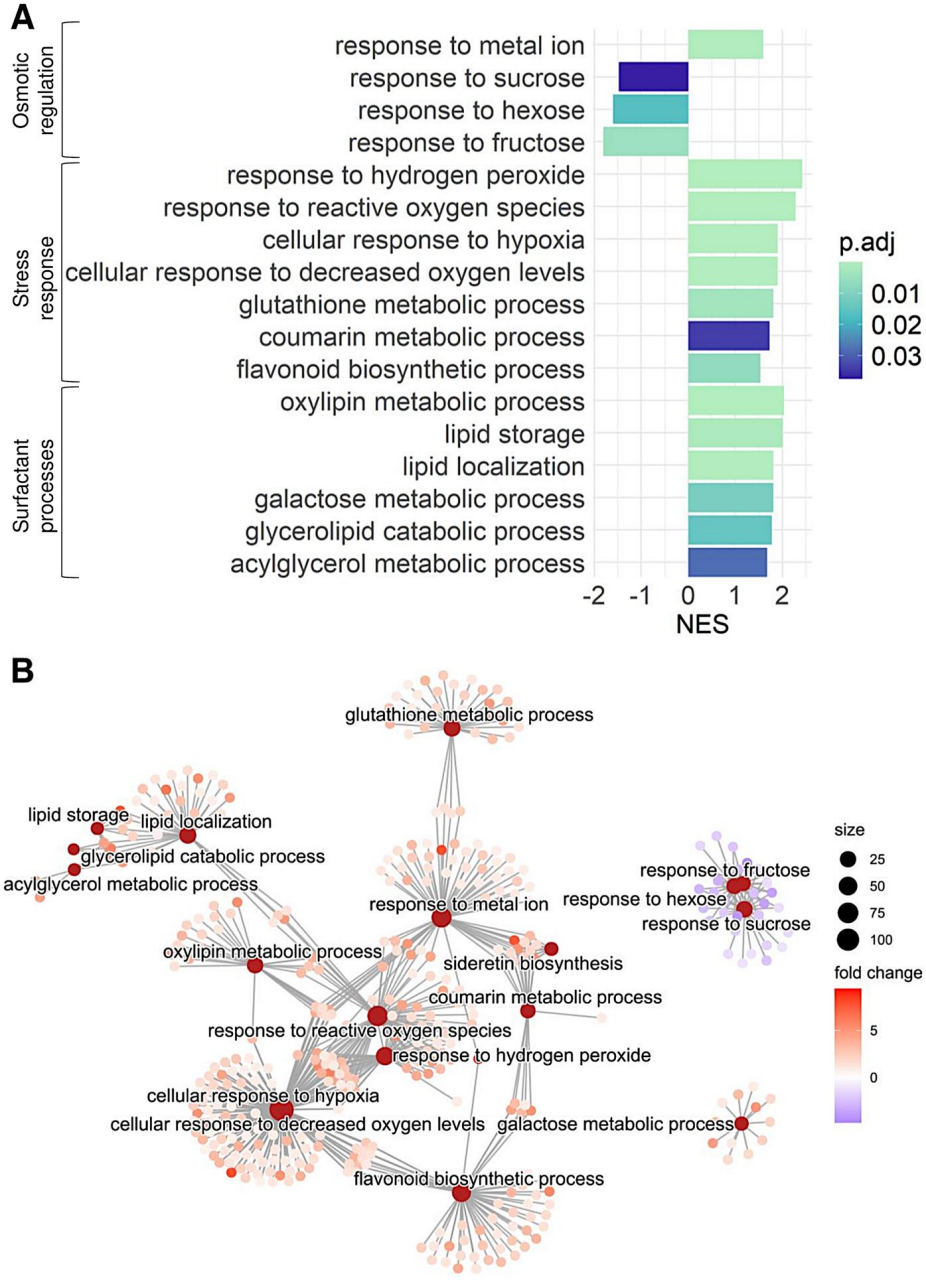
Gene set enrichment analysis results using the clusterProfiler 4.0 package in R v4.3.1 software (R Core Team 2022). (A) Histogram displaying the significantly enriched or depleted ontologies with potential roles in osmotic regulation, stress response, and surfactant processes in *E. grandis* . (B) Category net plot, displaying the clustering of the significantly enriched or depleted gene ontologies with a putative role in *E. grandis* embolism resistance and repair. Maroon nodes represent the gene ontologies, whereas the smaller nodes shaded from red to purple represent individual genes associated with the leading edge of the ontology. Node size is equivalent to the number of genes associated with the ontology, whereas the red to purple shading indicates the log_2_ fold change of the genes. The adjusted *p*‐value (*p*.adj) is on the basis of the Benjamini–Hochberg correction for multiple comparisons. The normalized enrichment score is depicted as NES.

In total, 263 gene ontologies were found to be significantly enriched or depleted after performing gene set enrichment analysis (Table S7). Upon critical inspection of the literature (Tomasella et al. 2020; Secchi et al. 2017, 2011; Feltrim et al. 2021; Schenk, Michaud, et al. 2021), 17 non‐redundant biological processes with the highest relevance to embolism sensing, prevention, and repair were identified (Figure 3). Several broad themes relating to the hydraulic response emerged from these categories, including osmotic regulation, stress response, and surfactant processes (Figure 3A). Among the stress‐related processes, responses to ROS, low oxygen levels, and secondary metabolite biosynthesis were upregulated during water stress. These stress‐related signals appear to have played a central role in triggering numerous biological processes relating to the storage, localization, and metabolism of lipids (Figure 3B). Interestingly, responses to monosaccharides appear to be depleted compared to controls and not linked to any stress responses, whereas glutathione metabolism, coumarin metabolism, and metal ion responses, enriched during drought, are linked to the stress response (Figure 3A,B).

### 3.3 Genetic Mechanisms for Cavitation Prevention

**FIGURE 4:**
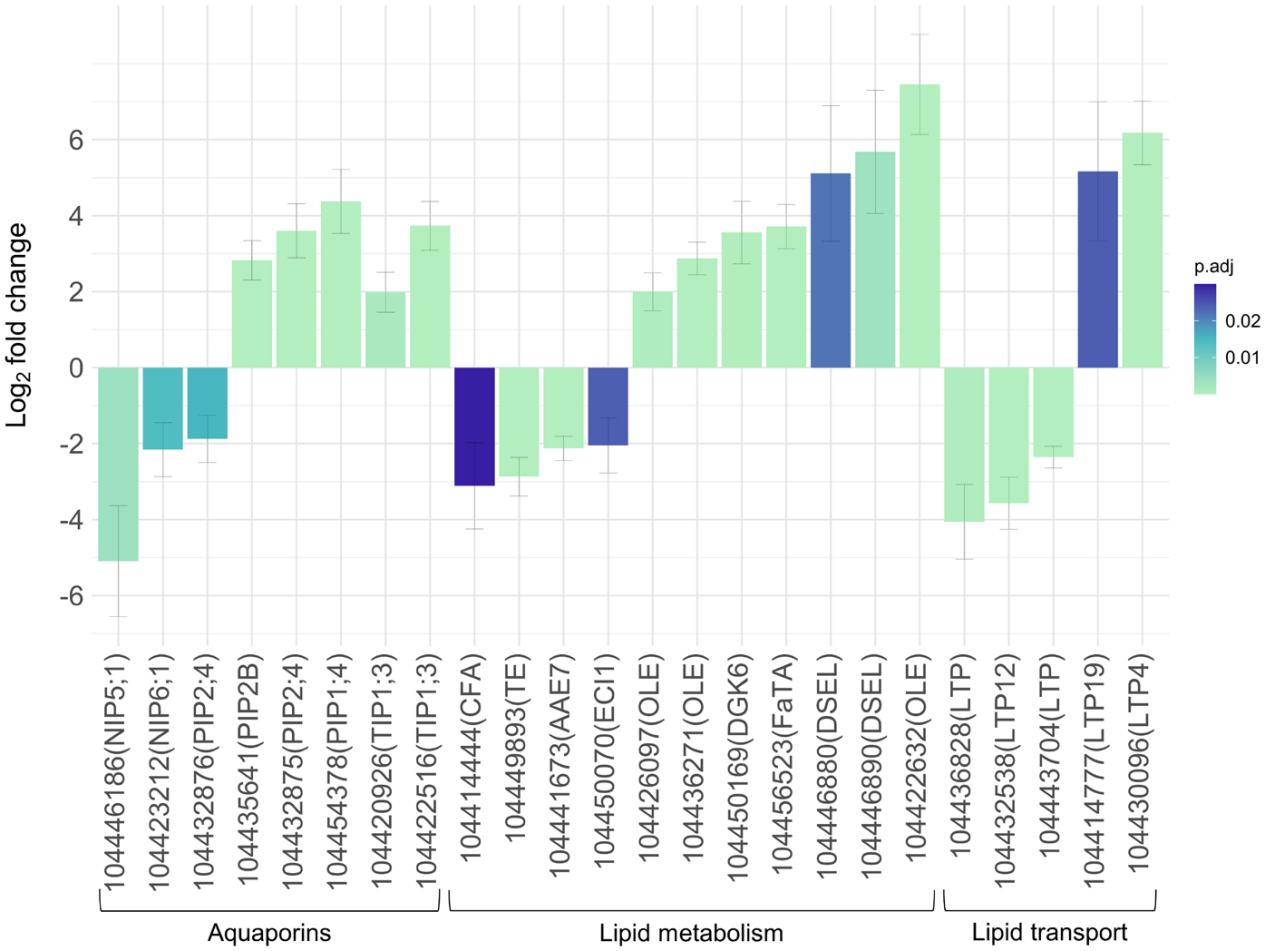
The expression of genes associated with lateral water movement and cavitation resistance in the xylem of *Eucalyptus grandis* . Upregulation of gene expression in the droughted condition is displayed as a positive log_2_ fold change, whereas a negative fold change indicates downregulation. Genes encoding for aquaporins, lipid metabolism and lipid transport are indicated. Benjamini–Hochberg correction was applied for *p*‐value adjustment.

Numerous gene expression candidates with putative roles in regulating the xylem water potential and suppressing nucleation of nanobubbles in the sap were identified (Table S8). For instance, genes encoding the aquaporins *PIP2B/2*;*4/1*;*4* and two isoforms of *TIP1*;*3* were upregulated during drought, as opposed to *NIP5*;*1/6*;*1* and *PIP2*;*4* that were downregulated (Figure 4). Expression of the lipid metabolic genes *cyclopropane‐fatty‐acyl‐phospholipid synthase* (*CFA*), *thioesterase* (*TE*), *acyl‐activating enzyme* (*AAE*) *7*, and *delta3‐delta2‐enoyl CoA isomerase* (*ECI*) *1* was lower compared to the controls. On the contrary, genes encoding three oleosin (*OLE*) family proteins, *diacylglycerol kinase* (*DGK*) *6*, *fatA acyl‐ACP thioesterase* (*FaTA*), and two alpha/beta‐hydrolase proteins (*DSEL*) were strongly induced during drought (Figure 4). Complementary to lipid metabolism, numerous transporters belonging to the LTP family were identified, with two nonspecific *LTPs* and *LTP12* displaying downregulation, and *LTP4/19* highly upregulated in drought‐stressed plants.

### 3.4 Priming for Hydraulic Recovery

**FIGURE 5:**
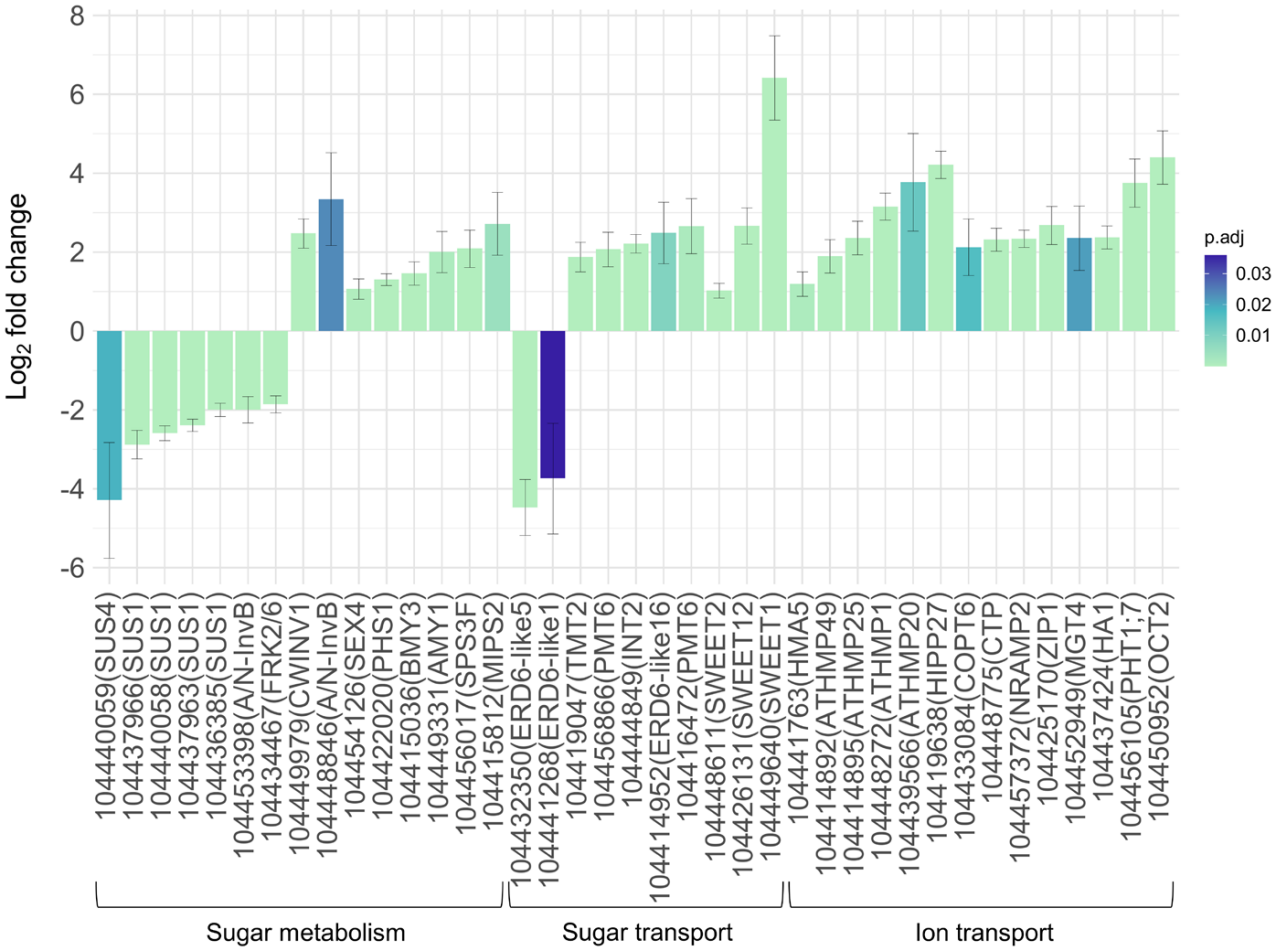
Gene sets associated with osmotic regulation in the xylem of *Eucalyptus grandis* . A positive log_2_ fold change indicates an upregulation in the droughted condition, whereas a negative fold change depicts downregulation. Genes that form part of the themes sugar metabolism, sugar transport, and ion transport are indicated. To correct for multiple comparisons, the Benjamini–Hochberg method was applied.

Genes with putative roles in osmotic regulation were identified in the differentially expressed dataset and categorized into sugar metabolism, sugar transport, and ion transport categories (Figure 5). During drought, sucrose catabolic genes including *sucrose synthase* (*SUS*) *1/4*, *alkaline/neutral invertase* (*A/N‐Inv*) *B*, and *fructokinase* (*FRK*) *2*;*6* were downregulated. Instead, sucrose and starch catabolism occurred through the upregulation of *cell wall invertase* (*CWINV*) *1*, *A/N‐InvB*, *starch‐excess* (*SEX*) *4*, *alpha‐glucan phosphorylase* (*PHS*) *1*, *beta‐amylase* (*BMY*) *3*, and *alpha‐amylase‐like* (*AMY*) *1* (Figure 5). The expression of *sucrose phosphate synthase* (*SPS*) *3F* and *myo‐inositol‐1‐phosphate synthase* (*MIPS*) *2* indicates elevated sucrose and myo‐inositol biosynthesis during drought. Transport of these sugars seemed to be largely facilitated by the expression of *early response to dehydration six‐like* (*ERD6‐like*) *1/5* in controls, whereas *Arabidopsis thaliana V‐PPASE* (*AVP*) *1*, *tonoplast monosaccharide transporter* (*TMT*) *2*, *polyol/monosaccharide transporter* (*PMT*) *6*, *inositol transporter* (*INT*) *2*, *ERD6‐like16*, and *sugars will eventually be exported transporters* (*SWEET*) *1/2/12* were upregulated in drier conditions. Ion transport was unanimously upregulated in droughted plants by genes encoding *heavy metal ATPase* (*HMA*) *5*, *heavy metal transport* (*ATHMP*) *1/20/25/49*, *heavy metal associated isoprenylated plant protein* (*HIPP*) *27*, *copper transporter* (*COPT*) *6*, *copper transport protein* (*CTP*), *NRAMP metal ion transporter* (*NRAMP*) *2*, *zinc transporter* (*ZIP*) *1*, *magnesium transporter* (*MGT*) *4*, *H[+]‐ATPase* (*HA*) *1*, *phosphate transporter* (*PHT*) *1*;*7*, and *organic cation/carnitine transporter* (*OCT*) *2* (Figure 5).

## 4 Discussion

### 4.1 Water Relations Influence Xylem Parenchyma Network Distribution During Drought

*Eucalyptus* species in general, and *E. grandis* in particular, naturally occur across a wide range of climatic conditions and thus display diverse water regulatory strategies (Gu et al. 2021; Li et al. 2019). Sustaining efficient water transport in the xylem is crucial to tree vigor, especially in eucalypts where embolism‐triggered hydraulic failure seems to be a primary mechanism for leaf and branch dieback (Li et al. 2018). According to our findings, copiously hydrated *E. grandis* displayed marginally negative water potentials and could thus freely transpire, whilst operating well within the margins of hydraulic safety (Choat et al. 2018). However, under drought, our plants implemented a more conservative water use strategy via stringent regulation of transpiration to mitigate excessive water loss and high xylem tension (Li et al. 2019). This response is an essential adaptive strategy to maintain the water potentials above the xylem cavitation threshold, as demonstrated in *Solanum lycopersicum sitiens‐flacca* double mutants unable to regulate transpiration (Brodribb et al. 2021). In our drought‐treated plants, transpiration declined gradually over time, while leaf water potential remained relatively steady at around −0.8 MPa. This stability suggests that the plants actively adjusted water use to avoid further declines in water potential. Under the conditions of this experiment, −0.8 MPa likely represents the water potential at which xylem function could be safely maintained. Interestingly, we observed an upward trend in the leaf water potential during the latter stages of the experiment that may have potentially coincided with the development of xylem features, such as smaller, more high‐density vessels and fibers, tailored towards hydraulic safety (Keret et al. 2024).

Studies of *E. tereticornis* and *E. sideroxylon* have demonstrated the capability of parenchyma to direct radial and circumferential water movement between tree tissues to reduce hydraulic tension induced by diurnal transpiration (Treydte et al. 2021). Upon exploring the parenchyma anatomy, we discovered that significantly smaller ray parenchyma and an increase in the number of rays occurred in response to the water deficit imposed (Table 1). These features will have improved the reach of parenchyma networks within the xylem of our drought‐treated plants, to facilitate effective delivery of water, sugars and ions to conduits experiencing excessively high water tension or embolism (Secchi et al. 2017). Furthermore, the delivery of solutes by vessel‐associated cells is largely dependent on a physical association with conduits via interconnected pits, and consequently elevated ray‐vessel contacts in combination with fewer isolated vessels may have served as a drought adaptive strategy in our plants (Słupianek et al. 2021). The results of the current study suggest that improving the effectiveness of water tension buffering or priming for refilling in *E. grandis* is, in part, reliant upon the increased presence of living cells within the xylem and the connectivity of vessel‐associated cells to the conduits (Secchi et al. 2017; Knipfer et al. 2019).

### 4.2 Stress‐Responsive Mechanisms Trigger Transcriptomic Remodeling

Given that parenchyma constitute the major metabolically active cell population in the xylem, most of the physiological, biochemical, and respiratory processes, underpinned by the expression of genes, occur within this fraction of the wood (Słupianek et al. 2021; Zhang et al. 2023). Parenchyma cells are essential role players in embolism prevention and recovery during drought, and exploration of the transcriptome has been an essential technique to identify the root mechanisms triggering this response (Secchi et al. 2011; Perrone et al. 2012). Many ontologies identified in the current study can be categorized into three broad groups, namely, stress response, surfactant processes, and osmotic regulation, which are all recognized candidates for hydraulic safety in the literature (Secchi et al. 2017). Interestingly, we identified that the core processes emerging during water stress gravitate around responses to ROS, hydrogen peroxide, hypoxia, and decreased oxygen levels (Figure 3A). Our findings do not support those of Secchi et al. (2011) that suggest a putative decrease in ROS formation and hypoxic stress occur as a consequence of embolism. It is well known that ROS do accumulate in the xylem of drought‐stressed trees, causing significant damage to organic substances within living cells, leading to cell death (Mantova et al. 2022). Since a viable parenchyma population is essential for stem water management, counteracting oxidative damage through antioxidant molecules was likely critical in our plants, as reflected by the enrichment of glutathione, coumarin, and flavonoid‐related ontologies (Knipfer et al. 2019; Nicolas‐Espinosa et al. 2023; Sultana et al. 2022; Rai et al. 2023). Together, these antioxidants help stabilize cellular membranes and also act as signaling molecules that modulate stress‐responsive pathways to maintain homeostasis under prolonged water deficit. Following the initial oxidative burst, controlled levels of ROS can also serve a signaling function (Huang et al. 2019), potentially triggering the mobilization of non‐structural carbohydrates, water redistribution, and cellular differentiation. Finally, since the transpiration stream is essential for adequate aeration of the living parenchyma cells, an embolism‐induced blockage could have triggered the hypoxic response observed in our droughted *E. grandis* plants (Manter and Kelsey 2008). The high level of interconnectedness of ROS and hypoxia to the surfactant and osmotic regulation themes in the category net plot suggests this as a triggering mechanism for a transcriptomic‐level response (Figure 3B). This study has demonstrated that transcriptomic remodeling induced by negative xylem tension clearly enriches ontologies associated with the transport or metabolism of surfactants, sugars, and ions, all of which are recognized candidates associated with cavitation prevention or priming for embolism refilling (Słupianek et al. 2021; Tomasella et al. 2020).

### 4.3 Aquaporin and Surfactant‐Mediated Cavitation Resistance

In response to the water stress, the expression patterns within the *E. grandis* transcriptome strongly suggest the activity of mechanisms designed to buffer negative xylem tensions, or alternatively prevent the nucleation and expansion of nanobubbles within the sap (Schenk, Michaud, et al. 2021; Treydte et al. 2021). Five aquaporins belonging to the PIP (*PIP2B/2*;*4/1*;*4*) and TIP (*TIP1*;*3*) families that possess water channel activity (Zwieniecki and Secchi 2017), were identified to be upregulated during drought (Figure 4). Intriguingly, the presence of these aquaporin families has been strongly implicated in lateral water movement under high vapour pressure deficit in *E. globulus* but not in *E. grandis* , possibly related to the lower water potentials observed in *E. globulus* (Feltrim et al. 2021). In contrast to this, our results indicate that sufficient drought pressure can trigger the differential expression of aquaporins in *E. grandis* xylem, most likely for radial or circumferential water movement (Figure 4). Consistent with our findings, PIP and TIP expression has been shown to increase in response to drought, particularly in vessel‐associated cells that operate at the forefront of water delivery to conduits (Zwieniecki and Secchi 2017).

Complementary to the buffering effect of aquaporins, lipids within the xylem sap may also serve as an embolism preventative mechanism in *E. grandis* (Schenk, Michaud, et al. 2021). Lipids function to coat hydrophobic or rough surfaces that serve as nucleation sites within the conduits and consequently enable water transport to occur under negative pressure while reducing the risk of embolism (Schenk et al. 2017). In our study, the presence of lipid metabolism (*CFA*, *TE*, *AAE7*, and *ECI1*) and transport (*LTPs*) genes in *E. grandis* receiving adequate hydration reiterates a fundamental requirement for these surfactants for safe sap transport, even under mild negative water potentials (Figure 1C) (Schenk, Michaud, et al. 2021). Recently, investigations of the lipidome in numerous angiosperm species revealed an abundance of galactolipids, phospholipids, and triacylglycerols in the xylem sap, which are postulated to play a role in coating nanobubbles (Huang et al. 2023). The gene expression patterns observed in our drought‐stressed *E. grandis* transcriptome indicate an increased presence of these lipids in the xylem under negative water potentials (Figure 4). For instance, we found that three *OLEs*, *FaTA*, and two *DSEL* isoforms that either promote triacylglycerol biosynthesis or inhibit its breakdown, respectively, to be upregulated (Ha et al. 2019; Sergeeva et al. 2021; Aznar‐Moreno et al. 2016). Our results indicate that an accumulation of triacylglycerol may be necessary for coating nucleation sites or alternatively for detoxification of toxic lipid intermediates accumulated during abiotic stress (Słupianek et al. 2021; Lu et al. 2020). Furthermore, the upregulation of *DGK6* in our drought treatment suggests an increase in phosphatidic acid that serves as a precursor for phospholipids and triacylglycerols, both of which are considered essential lipids in the nanobubble hypothesis (Huang et al. 2023; Schenk et al. 2017; Meringer et al. 2016; Siebers et al. 2016). Finally, an exceptionally high expression of *LTP4/19* in our drought‐affected *E. grandis* indicates that these are possibly the major transporters of lipids during drought, facilitating the unloading of lipids from vessel‐associated cells into conduits (Schenk, Michaud, et al. 2021).

### 4.4 Role of Osmoregulators for Priming Xylem Recovery

Despite employing some remarkable preventative measures, trees often experience excessively high water tension that can still lead to air seeding and subsequent embolism of the conduit (Saunders and Drew 2022). These conduits will require hydraulic recovery strategies that involve embolism sensing, the generation of an osmotic gradient, and, finally, water refilling (Tomasella et al. 2020). In this study, numerous genes (*SUS1/4*, *A/N‐InvB*, and *FRK2/6*) responsible for the breakdown of sucrose into simpler sugars such as fructose, glucose, or hexose were more highly expressed in the controls, presumably fueling metabolic pathways for the production of energy, complex carbohydrates, and metabolites (Stein and Granot 2019; Su et al. 2021; Wang et al. 2019; Pignocchi et al. 2021). Since embolism refilling relies upon the accumulation and subsequent efflux of sucrose from living parenchyma into the apoplast, the breakdown of sucrose within these cells would be counterproductive during drought (Secchi et al. 2021). Interestingly, we found that the invertases, *CWINV1* and *A/N‐invB*, that cleave sucrose into glucose and fructose were upregulated in our water‐stressed plants (Pignocchi et al. 2021; Peng et al. 2021). Studies on *Populus tremula × Populus alba* hybrids have demonstrated that invertases, including *CWINV1*, are essential for priming conduits for refilling by cleaving sucrose in the apoplastic domain, thus increasing monosaccharide accumulation in the cell walls (Pagliarani et al. 2019). This process reduces the osmotic potential of the conduits for refilling, as well as the sucrose concentration in the extracellular domain, thus further facilitating sucrose efflux from the living parenchyma (Secchi et al. 2021). To maintain a sucrose gradient in the parenchyma, the gene expression patterns in our transcriptome suggest that an elevated breakdown of starch occurred in the drought‐exposed plants, likely through the action of *SEX4*, *PHS1*, *BMY3*, and *AMY1* (McKinley et al. 2018; Thalmann and Santelia 2017; Feltrim et al. 2022; Lu et al. 2018). Starch breakdown is regarded as an essential factor in the embolism response and often correlates with an increase in sucrose within the parenchyma, suggesting that the free sugars released potentially serve as precursors to produce osmotica (Secchi et al. 2017). Consistent with these observations, we discovered that the expression of key regulators in sucrose (*SPS3F*) and myo‐inositol biosynthesis (*MIPS2*) was upregulated (Gao et al. 2019; Fleet et al. 2018; Souden et al. 2020). The expression of numerous sugar transporters in the xylem of our drought‐stricken *E. grandis* indicates that sugar mobilization may have been necessary for osmotic adjustment and the development of water gradients for embolism refilling (Secchi and Zwieniecki 2011). For instance, *TMT2*, *ERD6‐like16*, and *SWEET2* encode tonoplast‐localized transporters of sucrose, glucose, and fructose that, in conjunction with the plasma membrane‐localized *SWEET1*, could have regulated sugars within the vacuole and cytoplasm for osmotic adjustment during our water stress experiment (Saddhe et al. 2021; Dinant and Le Hir 2022; Mali et al. 2023; Sellami et al. 2019). Furthermore, the accumulation of myo‐inositol as an osmoprotectant has been observed in plants subject to drought, salt, or osmotic stress, and this was likely achieved by the action of transporters encoded by *PMT6* and *INT2* in our treated plants (Saddhe et al. 2021; Zhou et al. 2022; Doidy et al. 2019). During embolism recovery, sugar delivery from the phloem is also suggested to play a role in lowering the xylem water potential and thus facilitating refilling (Nardini et al. 2011). Our results indicate that the expression of *SWEET12* may be key to this mechanism in *E. grandis* , since this intercellular sucrose transporter has been shown to be localized in the parenchyma of both the xylem and phloem and thus may facilitate radial movement of sugars to prime embolized conduits for refilling (Hoffmann et al. 2022; Aubry et al. 2019; Mahboubi and Niittylä 2018). In addition to sugars, ions may also play a role as osmotica to elevate the driving force that draws water into the air‐filled conduits (Secchi et al. 2017). Interestingly, Secchi et al. (2011) postulated a role for metal ion transporters during refilling, of which numerous candidates responsible for the transport of Cu^2+^ (*HMA5*, *ATHMP20*, *COPT6*, and *CTP*), Cd^2+^ (*ATHMP25*), Fe^2+^/Zn^2+^ (*NRAMP2*), Mn^2+^ (*ZIP1*), and Mg^2+^ (*MGT4*) were identified to be upregulated in our drought‐stressed *E. grandis* (Li et al. 2017, 2020; Jogawat et al. 2021; Aprile et al. 2018; Urwat et al. 2021; Milner et al. 2013; Huang et al. 2016). Similarly, we found that transporters with an affinity for several metal ions, such as *ATHMP1/49* and *HIPP27*, were also highly expressed in response to the treatment (Li et al. 2020; Zhao et al. 2013). These essential micronutrients have been implicated in the formation of metalloenzymes that do indeed offer osmoregulatory functions in addition to protection against abiotic stress (Pandey 2018). Finally, transporters of ionic compounds such as protons (*HA1*), phosphates (*PHT1*;*7*), and cations (*OCT2*) displayed elevated expression in our drought experiments and may accumulate with sugars in non‐functional vessels to direct water flow from parenchyma (Słupianek et al. 2021; Secchi et al. 2021; Sun et al. 2018; Pinto and Ferreira 2015).

## 5 Conclusion

In the present study, we provide evidence in support of an adaptive parenchyma architecture and molecular response that function to buffer water tensions, and prime vessels for refilling during drought in *E. grandis* . Plants in our study displayed an acute ability to restrict transpiration rate, thereby reducing water potentials as an early response to water deficit. Upon prolonged exposure to these conditions, a higher frequency of rays in the xylem is produced to increase the reach of parenchyma networks. This architecture translated into an elevated number of ray‐vessel contacts with fewer isolated vessels, which likely enables efficient delivery of water and solutes. The reduced transpiration stream and air‐filled conduits likely trigger a molecular response centred around ROS and hypoxia. The expression of aquaporins may serve to buffer negative water potentials, while triacylglycerol biosynthetic and lipid transport genes could potentially facilitate sap flow under high tension by coating nanobubbles or hydrophobic surfaces to prevent nucleation. Our findings have revealed that numerous osmotica, namely sugars and ions, provide a priming mechanism for refilling, or alternatively lower water potentials to direct water flow. The *SWEET12*‐encoded transporter is likely a major facilitator of sucrose transport from the phloem to the xylem, thus providing an osmotic gradient for water delivery to conduits. This work highlights how ray architecture and parenchyma metabolic activity coordinate to sustain sap flow under water stress, offering a mechanistic link between wood anatomy and gene expression in the drought adaptation of *Eucalyptus*. Although further validation is required, this work highlights candidate mechanisms that could serve as markers of resilience. Future studies could explore these processes through protein localization assays, xylem sap metabolite profiling, and tissue‐level visualization of carbohydrate dynamics across species and environmental conditions.

## Conflicts of Interest

The authors declare no conflicts of interest.

## Supporting information


Data S1.

Data S2.

Table S1.

Table S2.

Table S3.

Table S4.

Table S5.

Table S6.

Table S7.

Table S8.

## Data Availability

All R scripts are available as supplementary data and on the platform GitHub in the repository https://github.com/Rafael‐Keret/Eucalyptus_transcriptomic_analysis. The raw sequencing data is available online under the BioProject accession PRJNA1012834 at the National Centre for Biotechnology Information (NCBI). The *Eucalyptus grandis* wood anatomical scans are freely accessible on Zenodo at https://doi.org/10.5281/zenodo.8246235 and https://doi.org/10.5281/zenodo.8245566.
