# Supplementary material for: Xylem Parenchyma Anatomy and Gene Expression Patterns Indicate Mechanisms of Cavitation Resistance in Eucalyptus grandis During Drought
Source: Plant Environ Interact. 2025 Jun 21;6(3):e70068. doi: 10.1002/pei3.70068 (PMC12181691; doi:10.1002/pei3.70068)
Supplement: Supplementary file 1 — Data S1. [file PEI3-6-e70068-s004.pdf]

Code S1: Impact of water deficit on the *Eucalyptus grandis* physiological response

Rafael Keret

2023-11-29

LEAF TRANSPIRATION

(1) Load packages

```
library("dplyr")
library("ggplot2")
library("tidyr")
library("emmeans")
library("car")
```

(2) Import Licor 600 dataframe

```
Licor_data <- read.table("./Data/Licor600/Table_S2_transpiration.csv", sep = ",", skip = 1,
                         header = T)
```

(3) Normality (Shapiro-Wilks)

```
Licor_data %>%
  group_by(Treatment, Day) %>%
  summarise(p.value = shapiro.test(log(Trans))$p.value) %>%
  print(n = 32)
```

(4) Calculate the mean transpiration and SE per day per treatment

```
T_mean <- Licor_data %>%
  group_by(Day, Treatment) %>%
  summarise(T_mean = mean(Trans),
            T_SE = sd(Trans)/sqrt(length(Treatment)))
```

(5) Perform a repeated measures ANOVA (time series data) and Tukeys post hoc
Set the Days as a factor since there was correlation between the predictor and intercept in the model.

```
Licor_data$Day <- factor(Licor_data$Day)
contrasts(Licor_data$Day) <- contr.sum(length(levels(Licor_data$Day)))
```

(6) Test the effect of treatment and days on transpiration
The dependent variable (transpiration) was square root (sqrt) transformed.

```
anova <- aov(sqrt(Trans) ~ Treatment * Day, data = Licor_data)
summary(anova)
```

(7) Test model assumptions

Normality

```
hist(residuals(anova))
plot(anova, which = 2)
```

Homogeneity of the variance: Residuals versus fitted values

```
plot(anova, which = 1)
leveneTest(residuals(anova) ~ Treatment, data = Licor_data)
```

(8) Tukeys HSD post hoc test, comparisons between treatments on the same day

```
Tukeys <- emmeans(anova, ~ Treatment | Day, contr = "tukey")

print(Tukeys)
```

(9) Extract the daily pairwise comparisons and adjusted p-values

```
Tukeys_post_hoc <- summary(contrast(Tukeys, interaction = "pairwise", adjust = "tukey"))
```

(10) Create a line plot with error bars

```
T_plot <- ggplot(T_mean, aes(x = Day, y = T_mean)) +
  geom_line(aes(linetype = Treatment, group = Treatment)) + geom_point(aes()) +
  geom_errorbar(aes(x = Day, ymin = T_mean - T_SE, ymax = T_mean + T_SE),
                width = 0.4, colour = "black", alpha = 0.9) +
  xlab("Day") + ylab(expression(Transpiration ~ (mmol~.m^-2~.s^-1))) +
  theme(panel.background = element_rect(fill = "white"),
        panel.grid.major = element_blank(),
        panel.grid.minor = element_blank(),
        axis.line = element_line(color = "black")) +
  theme(plot.margin = margin(3, 1, 1, 1, "mm")) +
  theme(axis.title = element_text(size = 17),
        axis.text = element_text(size = 17)) +
  theme(legend.title = element_text(size = 17),
        legend.text = element_text(size = 17)) +
  geom_text(data = T_mean %>% filter(Treatment == "C") %>%
              mutate(label = ifelse(Day == 0, "*", "***")),
            aes(label = label),
            vjust = -1.0, hjust = 0.45, color = "black", size = 5)
```

PRE-DAWN LEAF WATER POTENTIAL (PDLWP)

(1) Load packages

```r
library("dplyr")
library("ggplot2")
library("tidyr")
library("emmeans")
library("car")
```

(2) Import pre-dawn leaf water potential (PD_LWP) dataframe

```r
PD_LWP <- read.table("./Data/Physiology/Table_S3_pdlwp.csv", sep = ",", skip = 1,
                     header = T)
```

(3) Normality (shapiro-wilks)

```r
PD_LWP %>%
  group_by(Treatment, Day) %>%
  summarise(p.value = shapiro.test(log(PD_LWP))$p.value)
```

(4) Calculate the mean PD_LWP and SE per day per treatment

```r
LWP_mean <- PD_LWP %>%
  group_by(Day, Treatment) %>%
  summarise(LWP_mean = mean(PD_LWP),
            LWP_SE = sd(PD_LWP)/sqrt(length(Treatment)))
```

(5) Perform a repeated measures ANOVA (time series data) and Tukeys post hoc
Set the Days as a factor.

```r
PD_LWP$Day <- factor(PD_LWP$Day)
contrasts(PD_LWP$Day) <- contr.sum(length(levels(PD_LWP$Day)))
```

(6) Test the effect of treatment and days on pre-dawn leaf water potential (PD_LWP)
The dependent variable (PD_LWP) was square root (sqrt) transformed.

```r
anova <- aov(sqrt(PD_LWP) ~ Treatment * Day, data = PD_LWP)
summary(anova)
```

(7) Test model assumptions
Normality

```r
hist(residuals(anova))
plot(anova, which = 2)
```

Homogeneity of the variance: Residuals versus fitted values

```r
plot(anova, which = 1)
leveneTest(residuals(anova) ~ Treatment, data = PD_LWP)
```

(8) Tukeys HSD post hoc test, comparisons between treatments on the same day

```
Tukeys <- emmeans(anova, ~ Treatment | Day, contr = "tukey")

print(Tukeys)
```

(9) Extract the daily pairwise comparisons and adjusted p-values

```
Tukeys_post_hoc <- summary(contrast(Tukeys, interaction = "pairwise", adjust = "tukey"))
```

(10) Create a line plot with error bars

```
LWP_plot <- ggplot(LWP_mean, aes(x = Day, y = (-LWP_mean))) +
  geom_line(aes(linetype = Treatment, group = Treatment)) + geom_point(aes()) +
  geom_errorbar(aes(x = Day, ymin = -(LWP_mean - LWP_SE), ymax = - (LWP_mean + LWP_SE)),
              width = 0.4, colour = "black", alpha = 0.9) +
  xlab("Day") + ylab(expression(PDLWP~(MPa))) +
  theme(panel.background = element_rect(fill = "white"),
        panel.grid.major = element_blank(),
        panel.grid.minor = element_blank(),
        axis.line = element_line(color = "black")) +
  theme(axis.title = element_text(size = 17),
        axis.text = element_text(size = 17)) +
  theme(legend.title = element_text(size = 17),
        legend.text = element_text(size = 17)) +
  geom_text(data = LWP_mean %>% filter(Treatment == "C") %>%
             mutate(label = "***"),
            aes(label = label),
            vjust = -2.0, hjust = 0.45, color = "black", size = 5)
```

VOLUMETRIC WATER CONTENT ANALYSIS

(1) Load packages

```
library("dplyr")
library("ggplot2")
library("tidyr")
library("emmeans")
library("car")
```

(2) Import VWC dataframe

```
Logger <- read.table("./Data/LoggerCR1000x/Table_S1_vwc.csv", sep = ",", skip = 1,
                     header = T)
```

(3) Calculate the mean volumetric water content (VWC) and SE per day per treatment

```
VWC_mean <- Logger %>%
  group_by(Day, Treatment) %>%
  summarise(VWC_mean = mean(VWC, na.rm = TRUE),
            VWC_SE = sd(VWC, na.rm = TRUE)/sqrt(length(Treatment)))
```

(4) Perform a repeated measures ANOVA (time series data) and Tukeys post hoc
Set the Days as a factor.

```
Logger$Day <- factor(Logger$Day)
contrasts(Logger$Day) <- contr.sum(length(levels(Logger$Day)))
```

(5) Test the effect of treatment and days on volumetric water content (VWC)
The dependent variable (VWC) was square root (sqrt) transformed to achieve a normal distribution.

```
anova <- aov(sqrt(VWC) ~ Treatment * Day, data = Logger)
summary(anova)
```

(6) Test model assumptions
Normality

```
hist(residuals(anova))
plot(anova, which = 2)
```

Homogeneity of the variance: Residuals versus fitted values

```
plot(anova, which = 1)
```

(7) Tukeys HSD post hoc test, comparisons between treatments on the same day

```
Tukeys <- emmeans(anova, ~ Treatment | Day, contr = "tukey")

print(Tukeys)
```

(8) Extract the daily pairwise comparisons and adjusted p-values

```
Tukeys_post_hoc <- summary(contrast(Tukeys, interaction = "pairwise", adjust = "tukey"))
```

(9) Create a line plot with error bars

```
VWC_plot <- ggplot(VWC_mean, aes(x = Day, y = VWC_mean)) +
  geom_line(aes(linetype = Treatment, group = Treatment)) + geom_point(aes()) +
  geom_errorbar(aes(x = Day, ymin = VWC_mean - VWC_SE, ymax = VWC_mean + VWC_SE),
              width = 0.4, colour = "black", alpha = 0.9) +
  xlab("Day") + ylab(expression(VWC~(m^3~"/"~m^3))) +
  theme(panel.background = element_rect(fill = "white"),
        panel.grid.major = element_blank(),
        panel.grid.minor = element_blank(),
        axis.line = element_line(color = "black")) +
  theme(axis.title = element_text(size = 17),
        axis.text = element_text(size = 17)) +
  theme(legend.title = element_text(size = 17),
        legend.text = element_text(size = 17)) +
  geom_text(data = VWC_mean %>% filter(Treatment == "C") %>%
              mutate(label = "***"),
            aes(label = label),
            vjust = -0.45, hjust = 0.5, color = "black", size = 4)
```

(10) Test overall significance of the VWC over 30 days
Test normality

```
shapiro.test(VWC_mean$VWC_mean [VWC_mean$Treatment == "C"])
shapiro.test(VWC_mean$VWC_mean [VWC_mean$Treatment == "D"])
```

Non-parametric test (not normal)

```
wilcox.test(VWC_mean$VWC_mean [VWC_mean$Treatment == "C"],
            VWC_mean$VWC_mean [VWC_mean$Treatment == "D"],
            alternative = "two.sided")
```

PYRANOMETER READINGS

(1) Load packages

```
library("ggplot2")
library("tidyverse")
```

(2) Reading in only certain lines of the CSV

```
PYR <- read.table("./Data/LoggerCR1000x/Logger_Data_CR1000x.csv", sep = ",",
                  header = T)[5:2147, c(1, 3, 6)]
```

(3) Adding headings to the dataframe

```
colnames(PYR) <- c("TIMESTAMP", "PYR1", "PYR2")
```

(4) Separating the Dates and Times

```
PYR$TIMESTAMP <- strptime(PYR$TIMESTAMP, format="%Y-%m-%d %H:%M")
```

(5) Split into separate columns

```
PYR$Date <- as.Date(PYR$TIMESTAMP)

PYR$Time <- format(PYR$TIMESTAMP, "%H:%M")
```

(6) Converting LoggerNetData to numeric data types

```
str(PYR)

PYR[, c(2:3)] <- sapply(PYR[, c(2:3)], as.numeric)
```

(7) Filtering data based on date for experimental repeat 1

```r
PYR_E1 <- PYR %>% filter(Date >= as.Date('2021-04-23') & Date <= as.Date('2021-05-23'))
Repeat <- c("E1")
PYR_E1$Reps <- Repeat
```

(8) Filtering data based on date for experimental repeat 2

```r
PYR_E2 <- PYR %>% filter(Date >= as.Date('2021-07-13') & Date <= as.Date('2021-08-12'))
Repeat2 <- c("E2")
PYR_E2$Reps <- Repeat2
```

(9) Filtering data based on date for experimental repeat 3

```r
PYR_E3 <- PYR %>% filter(Date >= as.Date('2021-10-22') & Date <= as.Date('2021-11-21'))
Repeat3 <- c("E3")
PYR_E3$Reps <- Repeat3
```

(10) Join dataframes to create a single pyranometer table

```r
PYR_final <- rbind(PYR_E1, PYR_E2, PYR_E3)
PYR_final <- subset(PYR_final, PYR1 >= 10 & PYR2 >= 10)
```

(11) Calculate average PAR for the experiment within the tree canopy
Conversion of Pyranometer data from W/m2 to umole.m2/s requires a conversion factor x4.6 (i.e. 1 W/m2 = 4.6 umole.m2/s).
https://www.controlledenvironments.org/wp-content/uploads/sites/6/2017/06/Ch01.pdf.

```r
PYR_mean <- PYR_final %>%
  summarise(PYR1_mean = (mean(PYR1))*4.6,
            PYR1_SE = sd(PYR1),
            PYR2_mean = mean(PYR2)*4.6,
            PYR2_SE = sd(PYR2))
```

TEMPERATURE AND HUMIDITY

(1) Load packages

```r
library("dplyr")
```

(2) Specify path to iButton data files and append into a single dataframe for the repeats

```r
Path_to_csv <- dir("./Data/iButton",  pattern = ".csv", full.names = T)

iButton <- do.call(rbind, lapply(Path_to_csv, function(i) read.csv(i)))
```

(3) Calculate the mean temperature and humidity with SE

```r
abiotic_conditions <- iButton %>%
  summarise(Temp_mean = mean(Temperature),
            Temp_SE = sd(Temperature)/sqrt(length(Temperature)),
            Humid_mean = mean(Humidity),
            Humid_SE = sd(Humidity)/sqrt(length(Humidity)),)
```
