# Supplementary material for: Xylem Parenchyma Anatomy and Gene Expression Patterns Indicate Mechanisms of Cavitation Resistance in Eucalyptus grandis During Drought
Source: Plant Environ Interact. 2025 Jun 21;6(3):e70068. doi: 10.1002/pei3.70068 (PMC12181691; doi:10.1002/pei3.70068)
Supplement: Supplementary file 2 — Data S2. [file PEI3-6-e70068-s002.pdf]

Code S2: Determining the anatomical changes in the xylem parenchyma of *Eucalyptus grandis* subject to different watering regimes

Rafael Keret

2023-11-29

RAY AND AXIAL PARENCHYMA CELL AREA ANALYSIS

(1) Load packages

```
library(tidyverse)
library(ggpubr)
```

(2) Import QuPath raw csv data file and indicate tab ("/t") delimiter to separate the data into columns

```
Control_data <- read.table("./Data/input/Table_S4_control_WA.csv", sep = ",", skip = 1,
                           header = T)
Droughted_data <- read.table("./Data/input/Table_S5_drought_WA.csv", sep = ",", skip = 1,
                             header = T)

Control_data$Class <- gsub(": Positive", "", Control_data$Class)
Droughted_data$Class <- gsub(": Positive", "", Droughted_data$Class)
```

(3) Selecting Axial_parenchyma, Ray_parenchyma, Fibers and Vessels from the dataframe

```
Control_data <- subset(Control_data, Class %in% c("Axial_parenchyma", "Ray_parenchyma",
                                                  "Parenchyma", "Fibers", "Vessels"))
Droughted_data <- subset(Droughted_data, Class %in% c("Axial_parenchyma", "Ray_parenchyma",
                                                      "Parenchyma", "Fibers", "Vessels"))
```

(4) Calculate the mean cell area of ray and axial parenchyma per sample / slide

```
Control_mean <- Control_data %>%
  filter(Class %in% c("Axial_parenchyma", "Ray_parenchyma")) %>%
  group_by(Image, Class) %>%
  summarise(CA_Mean = mean(Cell..Area))

Control <- "C"
Control_mean$Treatment <- Control

Droughted_mean <- Droughted_data %>%
```

```
  filter(Class %in% c("Axial_parenchyma", "Ray_parenchyma")) %>%
  group_by(Image, Class) %>%
  summarise(CA_Mean = mean(Cell..Area))

Droughted <- "D"
Droughted_mean$Treatment <- Droughted
```

(5) Calculate the overall mean and se per treatment (i.e. Droughted vs Control)

```
Control_grouped <- Control_mean %>%
  group_by(Class) %>%
  summarise(CA_mean = mean(CA_Mean),
            CA_SE = sd(CA_Mean)/sqrt(36))

Control_grouped$Treatment <- Control

Droughted_grouped <- Droughted_mean %>%
  group_by(Class) %>%
  summarise(CA_mean = mean(CA_Mean),
            CA_SE = sd(CA_Mean)/sqrt(36))

Droughted_grouped$Treatment <- Droughted

Property_summary <- bind_rows(Control_grouped, Droughted_grouped, id = NULL)
head(Property_summary)
```

(6) Checking normality
Shapiro-Wilks test

Control

```
shapiro.test(Control_mean$CA_Mean [Control_mean$Class == "Axial_parenchyma"])
shapiro.test(log(Control_mean$CA_Mean) [Control_mean$Class == "Ray_parenchyma"])
```

Droughted

```
shapiro.test(Droughted_mean$CA_Mean [Droughted_mean$Class == "Axial_parenchyma"])
shapiro.test(log(Droughted_mean$CA_Mean) [Droughted_mean$Class == "Ray_parenchyma"])
```

(7) Significance / hypothesis testing

```
wilcox.test(Control_mean$CA_Mean [Control_mean$Class == "Axial_parenchyma"],
            Droughted_mean$CA_Mean [Droughted_mean$Class == "Axial_parenchyma"],
            alternative = "two.sided")

t.test(log(Control_mean$CA_Mean) [Control_mean$Class == "Ray_parenchyma"],
       log(Droughted_mean$CA_Mean) [Droughted_mean$Class == "Ray_parenchyma"],
       paired = FALSE)
```

(8) Axial and ray cell area graphs comparing treatments
Axial parenchyma graph

```
Axial_property <- subset(Property_summary, Class == "Axial_parenchyma")
```

CA

```
CA_AP <- ggbarplot(Axial_property, x = "Treatment", y = "CA_mean", fill = "Treatment") +
  ggtitle("Axial parenchyma") +
  theme(plot.title = element_text(hjust = 0.5, vjust = 2.5)) +
  ylab("CA"~"("*mu*M^2*")") + theme(strip.background = element_blank(),
                                    strip.placement = "outside") +
  theme(legend.position = "none") +
  geom_errorbar(aes(x = Treatment, ymin = CA_mean - CA_SE, ymax = CA_mean + CA_SE),
              width = 0.4, colour = "black", alpha = 0.9, size = 0.02) +
  scale_fill_manual(values = c("grey90", "grey50")) +
  theme(axis.text.x = element_text(colour = "black", size = 16, angle = 0,
                                   margin = margin(t = 3))) +
  theme(axis.text.y = element_text(colour = "black", size = 16, angle = 0,
                                   margin = margin(r = 3))) +
  theme(axis.title.x = element_blank()) +
  theme(axis.title.y = element_text(size = 16, margin = margin(r = 3))) +
  theme(text = element_text(family = "Aerial", size = 12)) +
  geom_signif(comparisons = list(c("C", "D")), map_signif_level = TRUE,
            annotations = c("NS"), y = 110)
```

Ray parenchyma graph

```
Ray_property <- subset(Property_summary, Class == "Ray_parenchyma")
```

CA

```
CA_RP <- ggbarplot(Ray_property, x = "Treatment", y = "CA_mean", fill = "Treatment") +
  ggtitle("Ray parenchyma") +
  theme(plot.title = element_text(hjust = 0.5, vjust = 2.5)) +
  ylab("CA"~"("*mu*M^2*")") + theme(strip.background = element_blank(),
                                    strip.placement = "outside") +
  theme(legend.position = "none") +
  geom_errorbar(aes(x = Treatment, ymin = CA_mean - CA_SE, ymax = CA_mean + CA_SE),
              width = 0.4, colour = "black", alpha = 0.9, size = 0.02) +
  scale_fill_manual(values = c("grey90", "grey50")) +
  theme(axis.text.x = element_text(colour = "black", size = 16, angle = 0,
                                   margin = margin(t = 3))) +
  theme(axis.text.y = element_text(colour = "black", size = 16, angle = 0,
                                   margin = margin(r = 3))) +
  theme(axis.title.x = element_blank()) +
  theme(axis.title.y = element_text(size = 16, margin = margin(r = 3))) +
  theme(text = element_text(family = "Aerial", size = 12)) +
  geom_signif(comparisons = list(c("C", "D")), map_signif_level = TRUE,
            annotations = c("*"), y = 160)
```

(9) Creating grid plots

```
library(cowplot)

plot_grid(CA_AP, CA_RP, labels = c('A', 'B'), label_size = 18, label_y = 0.99, ncol = 2)
```

RAY AND AXIAL PARENCHYMA PROPORTIONS

(1) Proportion of total parenchyma cell area per total cell area

```
Proportion_control <- Control_data %>%
  group_by(Image) %>%
  summarise(
    parenchyma_CA = sum(Cell..Area[Class %in% c("Axial_parenchyma", "Ray_parenchyma",
                                               "Parenchyma")]),
    total_CA = sum(Cell..Area[!(Class %in% c("Axial_parenchyma", "Ray_parenchyma",
                                            "Parenchyma"))])
  ) %>%
  mutate(prop_CA = (parenchyma_CA / (parenchyma_CA + total_CA)) * 100)

Proportion_droughted <- Droughted_data %>%
  group_by(Image) %>%
  summarise(
    parenchyma_CA = sum(Cell..Area[Class %in% c("Axial_parenchyma", "Ray_parenchyma",
                                               "Parenchyma")]),
    total_CA = sum(Cell..Area[!(Class %in% c("Axial_parenchyma", "Ray_parenchyma",
                                            "Parenchyma"))])
  ) %>%
  mutate(prop_CA = (parenchyma_CA / (parenchyma_CA + total_CA)) * 100)
```

(2) Calculating the overall proportional mean and SE per treatment

```
Proportion_C_grouped <- Proportion_control %>%
  summarise(Prop_mean = mean(prop_CA),
            Prop_SE = sd(prop_CA)/sqrt(36))

Proportion_C_grouped$Treatment <- Control

Proportion_D_grouped <- Proportion_droughted %>%
  summarise(Prop_mean = mean(prop_CA),
            Prop_SE = sd(prop_CA)/sqrt(36))

Proportion_D_grouped$Treatment <- Droughted

Proportion_summary <- bind_rows(Proportion_C_grouped, Proportion_D_grouped, id = NULL)
head(Proportion_summary)
```

(3) Checking normality
Shapiro wilks test

```
shapiro.test(sqrt(Proportion_control$prop_CA))
shapiro.test(sqrt(Proportion_droughted$prop_CA))
```

(4) Significance/hypothesis testing

```
t.test(sqrt(Proportion_control$prop_CA),
       sqrt(Proportion_droughted$prop_CA),
       paired = FALSE)
```

(5) Parenchyma proportion graph
Proportional CA

```
Prop_CA <- ggbarplot(Proportion_summary, x = "Treatment", y = "Prop_mean",
                     fill = "Treatment") +
  ggtitle("Parenchyma") +
  theme(plot.title = element_text(hjust = 0.5, vjust = 2.5)) +
  ylab("Proportional CA") + theme(strip.background = element_blank(),
                                  strip.placement = "outside") +
  theme(legend.position = "none") +
  geom_errorbar(aes(x = Treatment, ymin = Prop_mean - Prop_SE, ymax = Prop_mean + Prop_SE),
                width = 0.4, colour = "black", alpha = 0.9, size = 0.02) +
  scale_fill_manual(values = c("grey90", "grey50")) +
  theme(axis.text.x = element_text(colour = "black", size = 16, angle = 0,
                                   margin = margin(t = 3))) +
  theme(axis.text.y = element_text(colour = "black", size = 16, angle = 0,
                                   margin = margin(r = 3))) +
  theme(axis.title.x = element_blank()) +
  theme(axis.title.y = element_text(size = 16, margin = margin(r = 3))) +
  theme(text = element_text(family = "Aerial", size = 12)) +
  geom_signif(comparisons = list(c("C", "D")), map_signif_level = TRUE,
              annotations = c("NS"), y = 15.5)
```

RAY NUMBER AND VESSEL CONTACTS

(1) Read in data

```
Ray_vessel_contacts <- read.table("./Data/input/Table_S6_ray_vessels.csv", skip = 1,
                                  sep = ",", header = T)
```

(2) Calculating the overall mean and SE per treatment (i.e. Droughted vs Control)

```
Ray_grouped <- Ray_vessel_contacts %>%
  group_by(Treatment) %>%
  summarise(Ray_mean = mean(Ray_number),
            Ray_SE = sd(Ray_number)/sqrt(36))

Vessel_grouped <- Ray_vessel_contacts %>%
  group_by(Treatment) %>%
  summarise(Vessel_mean = mean(Vessel_contacts),
            Vessel_SE = sd(Vessel_contacts)/sqrt(36))
```

(3) Checking normality
Shapiro wilks test

Ray number

```
shapiro.test(Ray_vessel_contacts$Ray_number [Ray_vessel_contacts$Treatment == "C"])
shapiro.test(Ray_vessel_contacts$Ray_number [Ray_vessel_contacts$Treatment == "D"])
```

Ray-vessel contacts

```
shapiro.test(Ray_vessel_contacts$Vessel_contacts [Ray_vessel_contacts$Treatment == "C"])
shapiro.test(Ray_vessel_contacts$Vessel_contacts [Ray_vessel_contacts$Treatment == "D"])
```

(4) Significance/hypothesis testing
Ray number

```
t.test(Ray_vessel_contacts$Ray_number [Ray_vessel_contacts$Treatment == "C"],
       Ray_vessel_contacts$Ray_number [Ray_vessel_contacts$Treatment == "D"],
       paired = FALSE)
```

Ray-vessel contacts

```
t.test(Ray_vessel_contacts$Vessel_contacts [Ray_vessel_contacts$Treatment == "C"],
       Ray_vessel_contacts$Vessel_contacts [Ray_vessel_contacts$Treatment == "D"],
       paired = FALSE)
```

(5) Graphing
Ray number

```
RN_plot <- ggbarplot(Ray_grouped, x = "Treatment", y = "Ray_mean", fill = "Treatment") +
  ggtitle("Ray number") +
  theme(plot.title = element_text(hjust = 0.5, vjust = 2.5)) +
  ylab("Rays per ROI") + theme(strip.background = element_blank(),
                               strip.placement = "outside") +
  theme(legend.position = "none") +
  geom_errorbar(aes(x = Treatment, ymin = Ray_mean - Ray_SE, ymax = Ray_mean + Ray_SE),
                width = 0.4, colour = "black", alpha = 0.9, size = 0.02) +
  scale_fill_manual(values = c("grey90", "grey50")) +
  theme(axis.text.x = element_text(colour = "black", size = 16, angle = 0,
                                   margin = margin(t = 3))) +
  theme(axis.text.y = element_text(colour = "black", size = 16, angle = 0,
                                   margin = margin(r = 3))) +
  theme(axis.title.x = element_blank()) +
  theme(axis.title.y = element_text(size = 16, margin = margin(r = 3))) +
  theme(text = element_text(family = "Aerial", size = 12)) +
  geom_signif(comparisons = list(c("C", "D")), map_signif_level = TRUE,
              annotations = c("*"), y = 7.8)
```

Ray-Vessel contacts

```
RV_plot <- ggbarplot(Vessel_grouped, x = "Treatment", y = "Vessel_mean",
                     fill = "Treatment") +
  ggtitle("Ray-Vessel contacts") +
  theme(plot.title = element_text(hjust = 0.5, vjust = 2.5)) +
```

```r
  ylab("Ray-vessel counts") + theme(strip.background = element_blank(),
                                    strip.placement = "outside") +
  theme(legend.position = "none") +
  geom_errorbar(aes(x = Treatment, ymin = Vessel_mean - Vessel_SE,
                    ymax = Vessel_mean + Vessel_SE), width = 0.4,
                colour = "black", alpha = 0.9, size = 0.02) +
  scale_fill_manual(values = c("grey90", "grey50")) +
  theme(axis.text.x = element_text(colour = "black", size = 16, angle = 0,
                                   margin = margin(t = 3))) +
  theme(axis.text.y = element_text(colour = "black", size = 16, angle = 0,
                                   margin = margin(r = 3))) +
  theme(axis.title.x = element_blank()) +
  theme(axis.title.y = element_text(size = 16, margin = margin(r = 3))) +
  theme(text = element_text(family = "Aerial", size = 12)) +
  geom_signif(comparisons = list(c("C", "D")), map_signif_level = TRUE,
            annotations = c("**"), y = 12.3)
```

(6) Creating grid plots

```r
library(cowplot)

plot_grid(RN_plot, RV_plot, labels = c('A', 'B'), label_size = 12, label_y = 0.99, ncol = 2)
```

PROPORTION OF ISOLATED VESSELS

(1) Calculating the percentage of isolated vessels per slide / image (i.e. per tree sample)

```r
isolated_vessels <- Ray_vessel_contacts %>%
  group_by(Image, Treatment) %>%
  summarise(iso_percentage = (100 - (Vessel_contacts/Total_vessels)*100))
```

(2) Calculating the mean percentage of isolated vessels per treatment (i.e. Droughted vs Control)

```r
isolated_summary <- isolated_vessels %>%
  group_by(Treatment) %>%
  summarise(iso_mean = mean(iso_percentage),
            iso_SE = sd(iso_percentage)/sqrt(36))
```

(3) Checking normality
Shapiro-Wilks test

```r
shapiro.test(isolated_vessels$iso_percentage [isolated_vessels$Treatment == "C"])
shapiro.test(isolated_vessels$iso_percentage [isolated_vessels$Treatment == "D"])
```

(4) Significance / hypothesis testing
Non-parametric

```r
wilcox.test(isolated_vessels$iso_percentage [isolated_vessels$Treatment == "C"],
            isolated_vessels$iso_percentage [isolated_vessels$Treatment == "D"],
            alternative = "two.sided")
```
